# Supplementary figures and images for: Human Cytomegalovirus vMIA Inhibits MAVS Oligomerization at Peroxisomes in an MFF-Dependent Manner
Source: Front Cell Dev Biol. 2022 Apr 4;10:871977. doi: 10.3389/fcell.2022.871977 (PMC9014249; doi:10.3389/fcell.2022.871977)

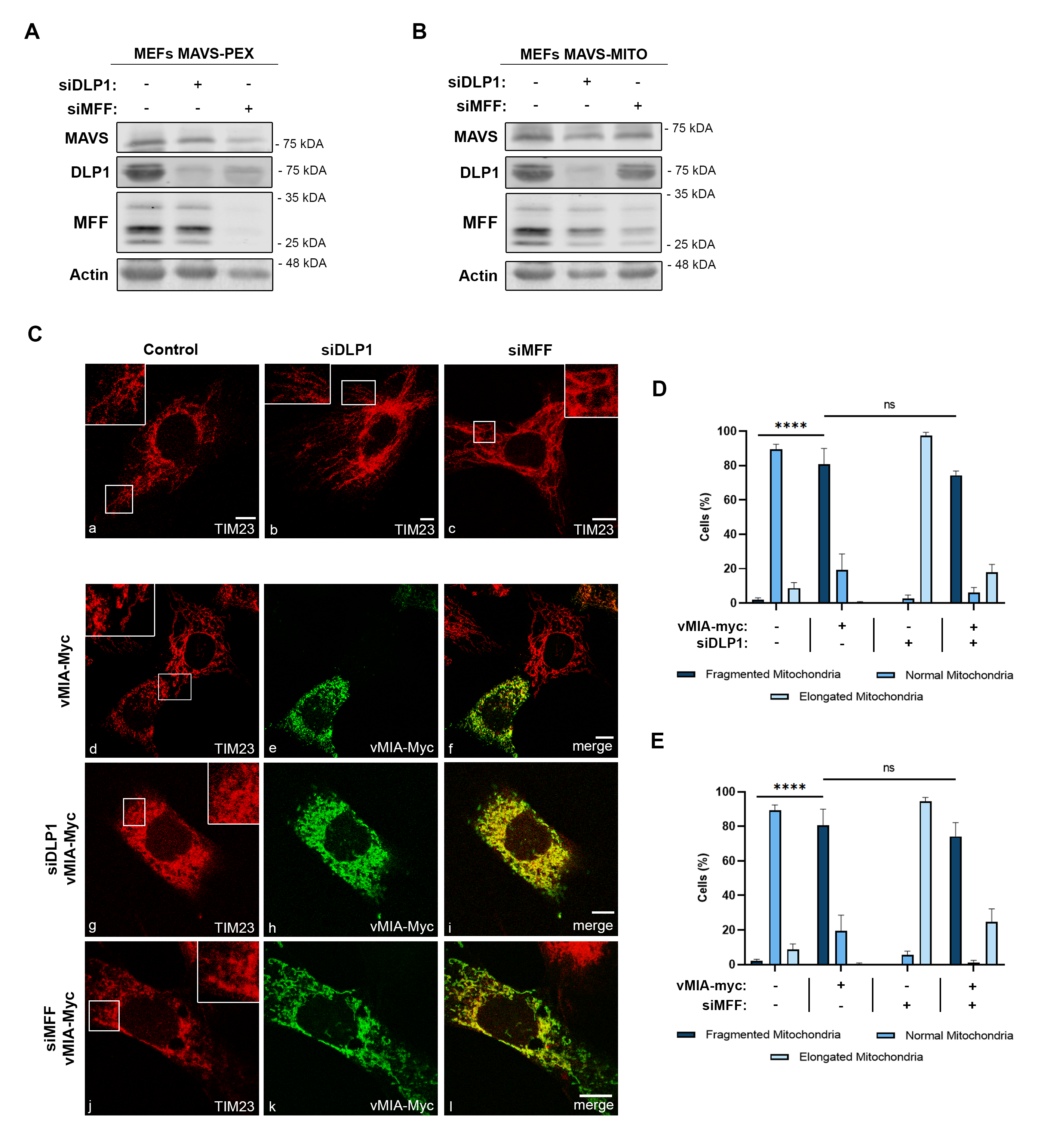

Supplement: Supplementary file 2 [file Image1.TIF]
